# Current Understanding on the Role of Standard and Immunoproteasomes in Inflammatory/Immunological Pathways of Multiple Sclerosis

**DOI:** 10.1155/2014/739705

**Published:** 2014-01-02

**Authors:** Elena Bellavista, Aurelia Santoro, Daniela Galimberti, Cristoforo Comi, Fabio Luciani, Michele Mishto

**Affiliations:** ^1^Interdepartmental Center for Studies on Biophysics, Bioinformatics and Biocomplexity “L. Galvani” (CIG), University of Bologna, 40126 Bologna, Italy; ^2^Department of Experimental, Diagnostic and Specialty Medicine (DIMES), University of Bologna, 40126 Bologna, Italy; ^3^Neurology Unit, Department of Pathophosiology and Transplantation, University of Milan, Centro Dino Ferrari, Fondazione IRCCS Cà Granda, Ospedale Maggiore Policlinico, 20122 Milan, Italy; ^4^Interdisciplinary Research Center of Autoimmune Diseases (IRCAD), University of Eastern Piedmont, “Amedeo Avogadro,” 28100 Novara, Italy; ^5^Inflammation and Infection Research Centre, School of Medical Sciences, University of New South Wales, Sydney, NSW 2052, Australia; ^6^Institut für Biochemie, Charité Universitätsmedizin Berlin, 10117 Berlin, Germany; ^7^Centro Interdipartimentale di Ricerca sul Cancro “Giorgio Prodi,” University of Bologna, 40126 Bologna, Italy

## Abstract

The ubiquitin-proteasome system is the major intracellular molecular machinery for protein degradation and maintenance of protein homeostasis in most human cells. As ubiquitin-proteasome system plays a critical role in the regulation of the immune system, it might also influence the development and progression of multiple sclerosis (MS). Both *ex vivo* analyses and animal models suggest that activity and composition of ubiquitin-proteasome system are altered in MS. Proteasome isoforms endowed of immunosubunits may affect the functionality of different cell types such as CD8^+^ and CD4^+^ T cells and B cells as well as neurons during MS development. Furthermore, the study of proteasome-related biomarkers, such as proteasome antibodies and circulating proteasomes, may represent a field of interest in MS. Proteasome inhibitors are already used as treatment for cancer and the recent development of inhibitors selective for immunoproteasome subunits may soon represent novel therapeutic approaches to the different forms of MS. In this review we describe the current knowledge on the potential role of proteasomes in MS and discuss the *pro et contra* of possible therapies for MS targeting proteasome isoforms.

## 1. Multiple Sclerosis and Proteasome Isoforms

Multiple sclerosis (MS) is a chronic disease of the central nervous system (CNS) characterized by the presence of inflammation, myelin damage, and axonal degeneration. There are two main clinical courses of multiple sclerosis: about 90% of MS patients experience the relapsing-remitting MS phase (RRMS), characterized by disability episodes followed by a complete or partial recovery. Multifocal lesions are found by magnetic resonance imaging, typically but not exclusively, in the white matter of the optic nerve, brain stem, cerebellum, and spinal cord. Some lesions are enhanced after intravenous administration of gadolinium, indicating breakdown of the blood-brain barrier (BBB) as a result of active inflammation. The majority of RRMS patients enter into a secondary progressive phase (SPMS), characterized by a variable degree of inflammation and a continuous and progressive neurological decline in disability state (with or without superimposed relapses) [1, 2]. A minor percentage (10%) of MS patients shows a primary progressive form of MS (PPMS), characterized by progression of neurological disability from onset. Clinically relevant factors differentiating RRMS and PPMS are age at disease onset (a decade later in PPMS) and gender (1 : 1.3 male/female in PPMS versus 1 : 2 in RRMS) [3]. Although the initial course of RRMS and PPMS is very different, both proceed at remarkably similar rates during the progressive phase. However, it is still an ongoing debate whether the RRMS and the progressive forms of MS are the same disease observed at different stages or whether they are pathogenetically different.

One of the factors characterising MS is the autoimmune response against self-antigens and the immune-mediated demyelination which contribute, at least in part, to the neurological manifestations. Based on scientific evidence, it has been proposed that a predisposing genetic background, in combination with environmental factors such as infection, diet, sun exposure, and smoking, drives the immune system to mount an immune response towards a yet unknown myelin antigen, eventually resulting in myelin disruption [4]. Indeed, genetic associations of HLA class II (HLA-DRB1*15) and HLA class I (HLA-A*02, -A*03, and -B*07) with MS, as well as the presence of autoreactive CD4^+^ and CD8^+^ T lymphocytes, together with other inflammatory cells and cytokines in active MS lesions, suggest an autoimmune pathogenesis [5, 6]. Several studies support the view that an immune response in MS subjects starts and is maintained in the periphery, and specifically in the lymphatic system, although the most lethal cytotoxic effect occurs in the brain with oligodendrocytes, neuron loss, and plaque formation (outside-in model) [2]. A competing view argues that the initial malfunction occurs within the CNS, similarly to other neurodegenerative diseases, by cytodegeneration, possibly focused on the oligodendrocyte-myelin complex, and a release of highly antigenic constituents that secondarily promote an autoimmune and inflammatory response in predisposed individuals [2, 7].

**Figure 1:**
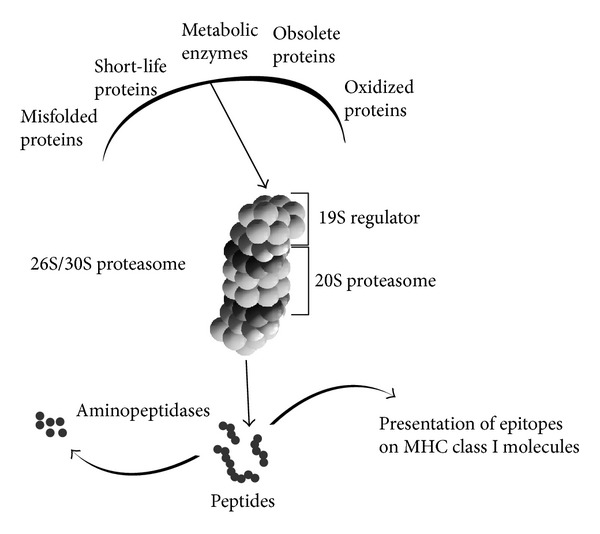
Schematic representation of the proteasome degradation pathways.

In the last few years, additional players have emerged in the MS pathogenic cascade, including proteasome and gut microbiota (for the latter see Section 3). The proteasome is the central catalytic unit of the ubiquitin-proteasome system, which plays several crucial functions for cell metabolism (Figure 1). By eliminating obsolete, misfolded, or aberrant proteins, the ubiquitin-proteasome system accomplishes housekeeping functions and maintains cellular homeostasis and the physiological levels of intracellular proteins. It has been demonstrated that proteasome inactivation leads to cellular death by apoptosis or necrosis [8–10]. The central role of ubiquitin-proteasome system in inflammatory responses is supported by evidence of its involvement in the on/off switching of many cellular pathways through the time-specific cleavage of short-life proteins, like transcription factors or molecules regulating the cell cycle [11]. Accordingly, the proteasome is crucial in several inflammatory processes by regulating cytokine signalling, cell proliferation, and clearance of potentially deleterious products of inflammation and is involved in the major histocompatibility complex (MHC) class I-mediated antigen presentation (Figure 1) [12]. Therefore, proteasome modulation can alter at different levels both the physiological and pathological processes of the immune system.

Different forms of proteasomes are known in eukaryotes. They vary in terms of catalytic subunits and regulatory complexes. The core 20S standard proteasome (s-proteasome) is a cylinder-shaped complex, that is, composed of four stacked rings, each consisting of seven protein subunits. Among them the *β*1, *β*2, and *β*5 subunits harbour the proteolytic active sites. The result of the association of the 20S proteasome core to the PA700 regulators is the 26S/30S proteasomes, which cleave polyubiquitylated proteins in an ATP-dependent manner. 20S proteasome can also bind the PA28 regulator, which alters proteasome catalytic activities [13, 14].

The immunoproteasome (i-proteasome) is an isoform of the 20S proteasome. It carries specific catalytic subunits, that is, *β*1i, *β*2i, and *β*5i (also known as LMP2, MECL-1, and LMP7, resp.), which confer to the i-proteasome quantitative differences in cleavage preferences and substrate degradation rates compared to the s-proteasome. I-proteasome is generally synthesized upon interferon-*γ* (IFN-*γ*) stimuli, but tumor necrosis factor-*α* (TNF-*α*) or lipopolysaccharide has also been found to be involved in its inducible expression [15, 16]. The vast majority of endogenous peptides that are presented by the MHC class I molecules at the cell surface and recognised by CD8^+^ T cells are generated by proteasomes. I-proteasome is generally linked to its high efficiency in the generation of the MHC class I-restricted epitopes. In support of this, i-proteasomes are predominantly expressed by professional antigen presenting cells (APCs), such as dendritic cells (DCs) and B cells, or in other cell types during inflammation, thereby indicating the i-proteasome as a major player of the MHC class I antigen presentation (Figure 1) [11, 17].

Preliminary observations on white and grey matter of MS patients suggested that the degradation rates of short fluorogenic peptides by 20S proteasomes are decreased when compared to brain-tissue controls [18]. These results, however, cannot be interpreted as a general decrease of the proteasome-mediated proteolytic activity, as recently shown in [19–21]. Furthermore, an accumulation of i-proteasome and its regulator PA28*αβ* has been observed in different cell types affected by MS, such as oligodendrocytes, astrocytes, macrophages/microglia, infiltrating lymphocytes, and weakly neurons [22]. Such disease-related expression of i-proteasome is in agreement with recent observations in the experimental model of MS, that is, the experimental autoimmune encephalomyelitis (EAE). In this model, the cerebral expression of i-proteasome and PA28*αβ* was increased as compared with baseline levels during the acute phase of EAE. Of note, i-proteasomes were also detected in neurons, infiltrated T lymphocytes, and microglia in EAE mice [23]. However, in this study by Zheng et al., an equal expression of s- and i-proteasome subunits has been described in control mouse brain, contrasting with other studies on rodents and humans which reported a faint expression of i-proteasome in young/adult brains [21, 24, 25]. Furthermore, Zheng et al. reported no differences in the i-proteasome expression by comparing young and old control mouse brains, which is in contrast to studies on other mammals such as rats [21, 26] and humans [27], but in agreement with a study conducted on nonhuman primates [28].

The expression of i-proteasome in MS lesions or in cells involved in MS mechanisms is important because this isoform has been recently linked to different inflammatory processes. Indeed, i-proteasomes are specifically implicated in cytokine-mediated inflammation, cell growth, and differentiation in mice [11]. I-proteasome depletion alters the T cell antigen receptor (TCR) repertoire formation, the number and differentiation of CD8^+^ T cells, and the production of proinflammatory cytokines [29]. In addition, i-proteasome depletion during IFN-*γ*-mediated oxidative stress is consistent with a deficient clearance of oxidized proteins and aggresomes [30, 31]. These events have been associated with worsening of EAE clinical score in *β*5i −/− mice [31], although discordant results have also been reported by others [32].

In the following sections we will discuss these and additional data suggesting an involvement of proteasomes in specific pathways underlying MS.

## 2. I-Proteasome and CD8^+^ T Cells in MS

CD4^+^ and CD8^+^ T lymphocytes reactive against myelin have been found in peripheral blood, cerebrospinal fluid (CSF), and CNS plaques of MS patients, but their role in MS pathogenesis is still a matter of debate. Antimyelin CD4^+^ T cells in MS have been widely studied because of their role in regulating cell-mediated inflammation, their ability in inducing EAE, and the identification of HLA-DRB1*15 allele as the most significant genetic risk factor associated with MS [33]. EAE can also be triggered by the administration of CD8^+^ T cells specific against myelin antigens in mice. In MS, CD8^+^ T cells exceed CD4^+^ T cells by 3–10-fold in regions of demyelination, and the degree of axonal damage within MS lesions correlates with the number of CD8^+^ T cells [33]. Furthermore, several studies described an increased prevalence of CD8^+^ cytotoxic T cells reactive against specific myelin epitopes in peripheral blood of MS patients compared to healthy controls [34–36]. These observations, in addition to the genetic associations of HLA class I alleles with MS risk, suggest an involvement of CD8^+^ T cells in MS [33].

Because i-proteasome is a major player in the processing of MHC class I-restricted epitopes in professional APCs or in inflamed conditions, it is likely that it is also involved in the presentation of myelin antigens in the MS brain. For instance, i-proteasome expression is induced in oligodendrocytes of MS patients [22]. These cells are the main producers of myelin, and hence likely to be the target of CD8^+^ T cells in MS. Indeed, CD8^+^ T cells were observed in close proximity to oligodendrocytes and demyelinated axons in brain tissue, towards which cytolytic granules were polarized [33]. The expression of i-proteasome in oligodendrocytes might therefore alter the presentation onto the MHC class I molecules of myelin antigens and the cytotoxic activity of specific CD8^+^ T cells towards these cells.

Although the abovementioned scenario lacks experimental validation, there is substantial support for this theory. For instance, our group has previously observed *in vitro* that i-proteasome carrying a polymorphic variant at codon 60 (i.e., HH60) of *β*1i subunit produces less amount of the myelin basic protein epitope MBP_111–119_ [22]. This epitope is presented on the HLA-A*02 molecule, although with moderate affinity [22] and memory CD8^+^ cytotoxic T cells specific for this epitope are more prevalent in the blood of MS patients than controls [35–37]. We also described a lower prevalence of the *β*1i HH60 variant among MS females with HLA-A*02^+^ genotype when compared to a matched control population. These observations led us to hypothesize that the lower risk of developing MS in HLA-A*02^+^ subjects carrying the *β*1i HH60 variant could be—at least in part—due to a lower production of MBP_111–119_ by oligodendrocytes or APCs in these subjects [22].

The key role of i-proteasomes in autoreactive CD8^+^ T cell response has been recently confirmed by the observation that mice lacking i-proteasome *β*5i-*β*2i subunits developed a multitissue autoimmune disorder mediated by CD8^+^ T cells *via* altered MHC class I-restricted self-antigen presentation [38]. The authors of the study speculated that a relatively high percentage of MHC class I molecules present “dangerous” epitopes in presence of inflammation and in the absence of i-proteasome. These self-peptides are low-affinity binders to the MHC class I complexes (as the epitope MBP_111–119_ [22]) and are better produced by s-proteasomes. Hence, in the absence of an appropriate i-proteasome activity these “dangerous” self-epitopes may be generated and targeted by autoreactive CD8^+^ cytotoxic T cells, thereby triggering an autoimmune response [38]. It is attractive to hypothesize that a similar mechanism is at work in MS and would imply that i-proteasome might hamper MS development by reducing the amount of “dangerous” self-peptides presented by APCs in periphery.

Another matter of debate relies on the mechanisms causing the disruption of the immune system tolerance and the activation of autoreactive CD4^+^ and CD8^+^ T lymphocytes towards CNS cells. Different studies suggest that molecular mimicry could be involved in the immune system disruption. This phenomenon describes the reaction of a single T cell clone to epitopes derived from both pathogen and human proteomes. It has been proposed that MS is triggered by a viral infection that, in the presence of (unknown) additional environmental and genetic factors, leads to an uncontrolled activation of autoreactive T cells. Such theory could explain in part the geographic distribution of the risk of developing MS [4] and is supported by several studies showing an increase of EBV-specific cellular immune responses in the blood and in the CSF of subjects with MS [5, 39–42], although the association with other viruses has also been found [4]. Conflicting results however exist about the role of molecular mimicry in driving pathological disorders associated with CD8^+^ T cells, as a comprehensive analysis on a broad range of CD8^+^ cytotoxic T cell clones showed a very limited number of cross-reactive T cells recognising both viral and self-epitopes [43].

**Figure 2:**
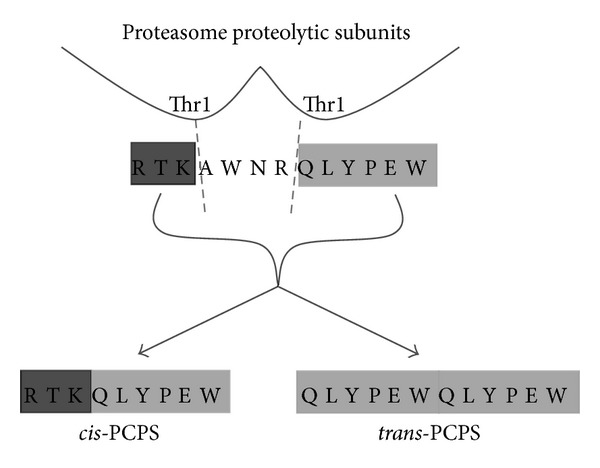
Proteasome-catalyzed peptide splicing (PCPS). PCPS can occur by ligation of two fragments of the same substrate molecule (*cis*-PCPS) or derived from two distinct protein molecules (*trans*-PCPS). Shown here are the representative cleavages (depicted by dotted lines) of the peptide gp100_40–52_ (sequence: RTKAWNRQLYPEW) by two distinct proteasome catalytic subunits, which generate the fragments RTK, AWNR, and QLYPEW. According to the PCPS model [44, 47], the protein is first cleaved by the active site residue Thr1 of the proteasome proteolytic subunits, thereby producing a protein fragment. The latter peptide stays attached to the catalytic centre where, subsequently, it is ligated to a second peptide generating the proteasome-generated spliced peptide.

The mechanisms of molecular mimicry related to CD8^+^ T cells in autoimmune disorders could be further investigated bearing in mind another proteasome-mediated process, named proteasome-catalyzed peptide splicing (PCPS). PCPS occurs through the binding of separate peptide fragments originating from a single protein, that is, *cis*-PCPS, or from two distinct protein segments, that is, *trans*-PCPS (Figure 2) [44–46]. The role of PCPS in MS has not been investigated yet, although it might be relevant for several reasons. Firstly, PCPS is more prone to generate MHC class I-restricted potential epitopes than the simple proteasomal peptide hydrolysis because of specific biochemical features of PCPS [47]. In addition, PCPS highly increases the diversity of MHC class I-restricted epitopes from self- and viral-antigens as the number of potential peptides presented on MHC molecules is several times higher than the number of peptides encoded in the proteome [48]. Consequently, through the PCPS there could be a significant increase of MHC class I-restricted epitopes with high sequence homology to viral and human proteomes. This phenomenon implies that the activation of CD8^+^ T cells specific for “spliced” viral epitopes with high or even complete homology with myelin antigens could represent a threat against myelin-producing cells and eventually take part in the development of MS.

## 3. Th17 Cells, Gut Microbiota, and Proteasome in MS

CD4^+^ T cells become activated by recognising antigens presented onto the MHC class II molecules, which are only expressed on professional APCs (such as DCs, macrophages, and B cells). Upon antigen stimulus, CD4^+^ T lymphocytes differentiate into two main subpopulations, T helper type 1 (Th1) cells and T helper type 2 (Th2) cells. Activated CD4^+^ T cells can also differentiate into regulatory T (Treg) cells, which are characterised by the expression of the forkhead box P3 (FoxP3) transcription factor [49].

More recently, a new T cell subpopulation, the Th17 cells, which secretes IL-17, IL-21, and IL-22, has been described and associated with the control of extracellular pathogens [50]. Th17 cells and their cytokines are associated with several autoimmune and inflammatory diseases, such as rheumatoid arthritis, systemic lupus erythematosus, MS, psoriasis, inflammatory bowel disease, allergy, and asthma [51]. In MS patients, IL-17 expression is increased in blood mononuclear cells and in CSF as well as at the site of lesions [52]. IL-17 and IL-22 promote blood-brain barrier permeability and CNS inflammation by inducing chemokine production in endothelial cells and by downregulating tight junction proteins. IL-17 also stimulates astrocytes to produce CXC chemokines that can attract neutrophils to the BBB and activate them to release vasoactive substances [53]. It has been shown that myelin-specific Th17 cells directly interact with neuronal cells in demyelinating lesions [54]. Either deficiency or neutralization of IL-17 delay the onset and reduce the severity of EAE [55]. Furthermore, IL-23 expands Th17 cells and is critical for the induction of EAE. In contrast, a recent paper reported that overexpression of IL-2 *in vivo* reverses EAE pathology by decreasing the Th1 and Th17 infiltration. Notably, under inflammatory conditions (such as in EAE), Th17 cells display plasticity because these cells can change phenotype in inflamed tissues and secrete proinflammatory cytokines such as IFN-*γ* instead of IL-17 [56].

A modifier of Th17 cell response in MS may be gut bacteria, which play an important role in shaping intestinal CD4^+^ T cell responses [57] and in affecting brain inflammation, as suggested by evidence on gut-brain communication [58, 59]. The mammalian gastrointestinal track harbors a highly heterogeneous population of microbial organisms, which vary across geographical areas and are essential for the complete development of the immune system. The gut microbes or “microbiota” also drive a swarm of T cell responses in the gut. For instance, segmented filamentous bacteria trigger intestinal Th17 cell responses; indeed when these bacteria are used to monocolonize germ-free mice they restore Th17 cell responses in the lamina propria of the small intestine [60]. Gut bacteria are also critically involved in the differentiation of some Treg cell subsets [61] as these specific microbial organisms have developed distinct ways to promote effector T cells or Treg cell differentiation in the gut [62]. The Treg/Th17 ratio and also the Treg cell frequency have been negatively correlated with MS severity [63], thereby suggesting that the measure of their balance could be an informative biomarker for evaluating or comparing the effectiveness of MS therapies.

In the context of MS models, it has been reported that the treatment of EAE mice with probiotics reduces neuroinflammation [64] and that different gut microbiota could induce [34, 65] or tackle CNS inflammation [66]. In addition, antibiotic-mediated depletion of the gut microbiota reduces the EAE severity and the levels of proinflammatory cytokines and chemokines, whereas it increases the levels of the anti-inflammatory cytokines IL-10 and IL-13. Moreover, IL-10-producing FoxP3+ Treg cells accumulate in the cervical lymph nodes of antibiotic-treated mice and protect *naïve* recipients against the transfer of EAE [65].

The tight connection between commensal gut microbiota, EAE, and Th17 lymphocytes has been recently investigated in two different models of EAE. Lee and colleagues [67] studied the induction of EAE by immunizing germ-free bacteria, specific-pathogen-free and control mice with MOG_35–55_ peptide + *Mycobacterium tuberculosis*. They observed that germ-free mice are highly resistant to EAE development and have a lower prevalence of Th17 and Th1 cells leading to the conclusion that there is a hampering of the systemic and neuronal proinflammatory Th17 and Th1 response during EAE in absence of commensal microbiota in mice. This phenomenon seems to be reversible because intestinal colonization with segmented filamentous bacteria in germ-free mice promotes EAE development. They concluded that the microbiota dynamically and reversibly impacts the programming of pathogenic immune response during autoimmunity and that microbial colonization may provide proinflammatory signals that affect the reciprocal development of Th and Treg cells both in gut and in CNS [67].

In a second article, Berer and colleagues [68] reported that germ-free mice develop less frequently EAE, a phenomenon accompanied by a reduced number of Th17 cells in the lamina propria and reduced secretion of IL-17 and IFN-*γ* by splenic T cells in response to cognate antigen stimulation. In this latter study, a spontaneous remitting-relapsing EAE mouse model has been used. These mice express, in a large proportion of their CD4^+^ T cells, a transgenic TCR that recognizes MOG_92–106_ peptide in the context of MHC class II molecules [68]. The fact that two independent studies described, in different models of EAE, an impairment of Th17-mediated induction of EAE in germ-free mice supports the hypothesis that gut microbiota may influence MS *via* Th17 cell activity.

Another modifier of the Th17 cell response in MS may be the i-proteasome. Indeed, it has been shown that the *in vitro* administration of i-proteasome *β*5i subunit inhibitor prevents the early activation of CD4^+^ T cells, their differentiation into Th17 cells, and the secretion of TNF-*α*, IL-23, and IL-6 [69]. *In vivo*, *β*5i inhibition or deficiency results in reduced Th1 and Th17 cell expansion and Treg cell development through STAT3/STAT1/SMAD phosphorylation [70]. The treatment with *β*5i subunit inhibitor also attenuates the progression of the experimental arthritis in mice [69]. Because this phenomenon acts on the Th17 differentiation pathway and it is not observed by inhibiting s-proteasome activity, we may speculate that a selective block of i-proteasome *β*5i subunit in mice might also tackle the development of EAE. Preliminary evidence in dextran sodium sulfate-induced colitis indirectly support such speculation, since this animal model mimics inflammatory bowel diseases, such as Crohn's disease and ulcerative colitis, which are characterized by a marked mucosal infiltration of T cells that secrete Th1 and Th17 cytokines and alterations of faecal and mucosal bacterial communities [71]. Interestingly, in *β*5i subunit −/− mice and in wild type mice treated with a proteasome inhibitor, there is a reduction in the secretion of proinflammatory cytokines and chemokines, the infiltration into the colon by neutrophils, and the expansion of Th1 and Th17 cells, thereby preventing excessive tissue damage [72]. These observations are in agreement with the results of Basler et al. [73], which showed a role of *β*5i subunit inhibition in reducing the production of proinflammatory cytokines, inflammation, tissue destruction, and consequently pathological symptoms of experimental colitis.

These data suggest that in EAE the activity of Th17 cells could be regulated by gut microbiota and i-proteasomes. Therefore, both of them may be potential targets for the treatment of MS, although there are no studies that investigated the direct interaction between gut microbiota and i-proteasome in EAE.

## 4. Humoral Immunity, Proteasomes, and MS

The understanding of MS pathogenesis has been mostly driven by studies on T cells and their inflammatory cytokines produced in damaged tissues [74]. The interest regarding the antibody-dependent as well as antibody-independent B cell involvement has received a strong boost from the success of clinical trials targeting B cells in MS and other autoimmune diseases [75, 76].

Beyond their ability to produce antibodies, B cells function as APC, thereby contributing to T cells activation in the CNS [77]. They also influence the immune response through the production of effector cytokines, such as those involved in immune regulation (e.g., anti-inflammatory IL-10), polarization (IL-4), and cytokines involved in lymphoid tissue organization (e.g., TNF-*α* and leukotrienes) [78]. Remarkably, decreased levels of IL-10 and increased concentrations of TNF-*α* and leukotrienes have been described in patients affected by MS [79], thus contributing to abnormal T-cell activation. This fact provides a conceivable mechanism of action to explain why B cell depletion may be relevant, both in the periphery and in the CNS, in diminishing new MS activity [79]. Indeed, Rituximab, a monoclonal antibody against CD20 molecules, exerts its therapeutic effect through a rapid and profound depletion of peripheral B cell, along with a significant reduction in the volume of T2 lesions and clinical relapse in the RRMS patients, and a reduced disease progression in PPMS [80, 81]. Additionally, in a small cohort of PPMS, it has been shown that Rituximab temporarily suppresses the activation of B cells in CSF [82]. However, the presence of regulatory B cell subsets (B regs), which could either induce or inhibit immune response, accounts for the variable effects that targeting B cells may have *in vivo* [77–80]. At present, new monoclonal antibodies (i.e., Ocrelizumab, Ofatumumab) targeting CD20 or specific surface markers of B cell subset (i.e, Atacicept) are under investigation in phase II/II trials [83, 84].

Although at present there is no data available on proteasome isoforms, B cell regulation, and MS, the recent observation of Hensley et al. [85] is relevant to connect all these three topics. Indeed, the authors reported that i-proteasome *β*1i subunit −/− mice have a defect in B cells maturation and Ig isotope switch upon viral infection as well as in CD4^+^ T cell survival and DC activation. They identified in the NF-*κ*B activation one of the pathways affected by the presence of intermediate type proteasomes instead of the i-proteasome, which is normally present in these cells. A role of i-proteasome in modulating NF-*κ*B signalling has also been observed by Maldonado and coworkers [86] in retinal pigment epithelial cells of *β*1i subunit −/− mice. In knockout mice a higher content and a diminished activation of the NF-*κ*B alternative pathway, as well as a delayed termination of the classical pathway, after *in vitro* stimulation by TNF-*α*, has been observed compared to wild type littermates [86].

Concerning the role and significance of antibodies in MS patients, the presence of CSF oligoclonal bands and increased immunoglobulin IgG synthesis is a frequent feature of MS [87] as well as other localized autoimmune diseases of the CNS [88]. These pathogenic autoantibodies (autoAbs) can induce tissue damage and thus be involved in plaque initiation and demyelination by recruiting macrophages and by complement deposition in white matter lesion of MS patients [89]. However, the antigenic targets of these antibodies and their potential use as biomarkers of MS are still a matter of debate. Indeed, autoAbs against antigens not specific for the CNS have also been associated with MS, although it is unclear if they are pathogenic effectors instead of being secondary products of the release of antigens upon CNS tissue damage. Proteasome Abs, for example, are elevated in sera of RR-, PP-, and SP-MS patients compared to other autoimmune diseases or healthy controls [90–92]. It has been shown *in vitro* that autoAbs against 20S proteasome block the proteasome activation by PA28 regulator, thereby suggesting that these autoAbs might have a regulatory function towards extracellular proteasomes such as circulating proteasomes [93]. Notably, although proteasomes are mainly studied as intracellular proteases, extracellular circulating proteasomes are normally present in peripheral blood, and their levels are significantly increased in a variety of pathological conditions, including autoimmune diseases and tumours [94]. In particular, as biomarkers of ongoing pathological mechanisms, circulating proteasomes have demonstrated to have prognostic power as regards therapy outcome and survival in multiple myeloma patients [95]. Although cells originating extracellular proteasomes detected in peripheral blood and in the CSF have not been identified, an active release of circulating proteasomes has been recently proposed [96] as they have been copurified with exosomes [97]. In line with this hypothesis, the immunological activity rather than the cellular damage has been suggested as the causative mechanism for increased circulating proteasome levels in sepsis and sever injury [98]. Recently, a preliminary study carried out on a limited number of patients affected by RRMS has shown that circulating proteasome amount increases in MS and even further in MS patients treated with IFN-*β*. The authors have also described a specific proteasome activity pattern in plasma of MS patients although they have not reported appropriate control experiments with proteasome inhibitors [99]. This preliminary observation, however, might be relevant for future studies. Indeed, a fascinating speculation is that circulating proteasomes in peripheral blood are not only simple biomarkers of inflammatory status, but also active proteases that might control cytokine levels, cell-mediated cytotoxicity, and plasma membrane permeability [94] and synergize with other component to ameliorate tissue damage [97].

## 5. Maintenance of Cellular Homeostasis during Inflammation-Mediated Oxidative Stress in MS

The pathological mechanisms of neurodegeneration, although largely unknown, are often mediated by oxidative stress and excitotoxicity (degenerative cascade), two processes that are closely interactive [100, 101]. The increased production of reactive oxygen and nitrogen species induces oxidative damage to different cellular components including lipid, DNA, and proteins [102]. Accordingly, in MS patients, oxidized DNA is present in a small number of reactive astrocytes as well as in oligodendrocyte nuclei, with evidence of apoptosis [103]. Similarly, lipid peroxidation-derived structures (malondialdehyde and oxidized phospholipid epitopes) can be detected in the cytoplasm of oligodendrocytes and some astrocytes as well as in degenerating neurons within grey matter lesions [103]. Oxidized proteins are more prevalent in cerebellar astrocytes as well as in spinal cord neurons of EAE mice [104, 105]. In such scenario, an effective removal of oxidized proteins seems to be a key element to maintain cellular homeostasis during neuroinflammation.

Studies performed on neuronal cell lines have suggested that proteasome plays a central role in mitochondria homeostasis. Proteasome inhibition decreases the activity of complexes I and II and increases the production of reactive oxygen species and the accumulation of lipofuscin, a highly oxidized cross-linked aggregate of oxidized protein and lipid [106, 107]. In addition, proteasome is essential in maintaining cell homeostasis by degrading obsolete, damaged, and oxidized proteins [108–112]. Notably, the 20S proteasomes are more resistant to oxidative stress than 26S proteasomes and seem to be able to degrade oxidized proteins in an ATP-independent manner [113, 114]. Furthermore, i-proteasome expression is induced during oxidative stress in several inflammatory-based diseases in the CNS and in peripheral organs [30, 115, 116] and it provides enhanced cellular resistance to oxidative stress, at least in part by an increased degradation rate of oxidized proteins compared to s-proteasome [117]. Indeed, the blocked expression of *β*1i subunit by siRNA significantly reduces the adaptive response to mild oxidative stress in mouse embryonic fibroblasts [116], *β*5i-depleted retinal pigment epithelial cell viability is more compromised than wild type cells [30], and *β*1i subunit −/− mice exhibit higher levels of protein carbonyls in brain and liver upon aging than those of their wild type littermates [118]. Accordingly, Seifert and coworkers [31] have shown an accumulation of oxidized and polyubiquitylated proteins and aggresome-like induced structures upon INF-*γ* stimuli in the liver and brain of i-proteasome *β*5i subunit −/− mice. Moreover, *β*5i subunit deficient cells and tissues are not only more sensitive to apoptosis but also have a delayed activation of NF-*κ*B after TNF-*α* stimulation [31]. This dependence of protein oxidation clearance on i-proteasome activity might be pivotal for MS because i-proteasome *β*5i subunit −/− mice showed an earlier onset and worse clinical score than wild type mice in an EAE model [31] although this fact, recently, has been disputed by Nathan and colleagues [32].

Overall, these results suggest that i-proteasomes may influence onset and progression of MS by affecting the response of different cell types to the inflammatory aggression in the CNS.

## 6. Is Proteasome Inhibition a Potential Therapy of MS?

The administration of immunomodulatory drugs (glatiramer acetate and IFN-*β*) represents the first line therapy for RRMS, but these drugs are seldom useful towards the progressive form of MS [119]. The partial or total inefficacy of the common MS treatments in SPMS and PPMS patients demands the identification of novel therapies. The progressive forms of MS seem to be characterized by peculiar immunological mechanisms that differ from RRMS. In PPMS and SPMS the whole brain is affected and inflammation as well as axonal injury is diffuse, whereas in RRMS inflammation and tissue damage are more focalized in plaques [120]. In the progressive forms of MS, the CD4^+^ and CD8^+^ T cells and the B cells seem to be part of the pathological mechanisms, although with characteristics that differ from those observed in RRMS and without a clear correlation between immune cell activation and clinical measures of disease duration and severity, especially in PPMS [121]. Considering the complex pathogenic mechanisms at the basis of MS development, further studies would be needed to better characterize the role of different immune system players, including proteasomes, autoAbs as well as specific Th17 and CD8^+^ T cells, in the different forms of MS. These studies are likely to support the discovery of new diagnostic and prognostic biomarkers for different MS forms and to generate novel therapeutic drugs such as the specific proteasome inhibitors. Indeed, proteasome inhibitors have been utilised as therapeutic approach towards other diseases, such as multiple myeloma, and selective inhibitors for s- or i-proteasome have been recently developed [122].

Two factors could influence the success of novel therapies based on proteasome inhibitors: their toxicity profiles and their delivery pathways to the CNS and/or the periphery. Regarding the former, the experience of the first proteasome inhibitor, named Bortezomib, approved for clinical treatment of hematologic malignancies, showed that the toxicity could be a limiting factor [122]. However, this disadvantage can be controlled with new inhibitors specific for i-proteasome subunits that can therefore block proteasome activity only in specific cells or pathological conditions [122]. In such contest, the induction of i-proteasome expression in specific cell types upon MS onset—reviewed in Section 1—is a pivotal element ought to be borne in mind.

An additional critical issue is drug delivery. Indeed, the inhibition of i-proteasome is detrimental in tackling the oxidative stress during inflammation, leading to the accumulation of oxidised proteins [11]; this has been linked to the disputed observation that the depletion of *β*5i subunit anticipated EAE onset [31, 32] (Table 1). However, further investigations have to be performed since the blockage of i-proteasome activity resulted in a decreased expression of inflammatory biomarkers in *ex vivo* analyses of microglia of a mouse model of Alzheimer's disease [19]. Conversely, an inhibition of i-proteasomes limited to the periphery and towards immune system components such as B and Th17 lymphocytes might be beneficial in treating MS. Noteworthy, the promising results of the clinical trials with the monoclonal antibody Rituximab for the treatment of MS (see Section 4) are consistent with the hypothesis that also a depletion of B cells might ameliorate MS disease. In mice, such depletion could be achieved by a defect in *β*1i subunit expression [85] (Table 1).

Furthermore, Th17 cells could be targeted for ameliorating MS course. As i-proteasome inhibition decreases the activation of Th17 cells in mice [69, 70], it can be envisaged that i-proteasome inhibitors could be used to limit Th17 cell activation and EAE progression in mice (Table 1). The first test of this hypothesis could be obtained by treating EAE mice with inhibitors of the i-proteasome *β*5i subunit, as it has been already done for other experimental disease models [69, 73]. Notably, a blockage of the i-proteasome activity along the Th17 cell pathway could be coupled to the therapeutic administration of probiotics (live beneficial bacteria) or prebiotics (compounds that stimulate the growth of beneficial bacteria) in EAE mice, given their common action on Th17 lymphocytes [67, 68]. Nonetheless, whether the modulation of gut microbiota could have similar beneficial effects also on MS is largely unknown. In EAE, the depletion or the strong modification of gut microbiota showed beneficial effects on the development of the disease [67, 68]. However, unlike mouse models, the human being has a broad variety of diet, environment, genetics, and early microbial exposure features that lead to highly diversified microbiota, which is furthermore extremely adaptable and variable over time [125, 126]. Therefore, the identification of a beneficial or detrimental microbiota towards MS might be strenuous.

While the potential inhibition of i-proteasome activity in B and Th17 cells points towards a beneficial effect against MS, the knowledge of the role of circulating proteasome and of proteasome Abs remains poor. Because of high levels of circulating proteasomes and proteasome Abs in the serum of MS patients [90, 99] a tempting speculation is that the production of proteasome Abs might aim to affect the circulating proteasome activity, although the role of circulating proteasomes in MS and more in general in the peripheral blood is largely unknown (Table 1). Further studies are mandatory to investigate such an issue because a therapy with proteasome inhibitors delivered through peripheral blood would immediately affect circulating proteasomes.

The potential effects of an i-proteasome inhibition within the CD8^+^ T cell-mediated immune response are still unclear. This inhibition could affect therapy outcome depending on whether the drug is delivered in the periphery only or also in the CNS. Indeed, i-proteasome could influence the presentation of endogenously produced myelin antigens in oligodendrocytes (i.e., in CNS) and in bone marrow-derived APCs (i.e., in periphery) [123, 127], although the outcome of the activation of antimyelin CD8^+^ T cells is still a matter of debate. For instance, in transgenic mice the induction of EAE by HLA-A*03-restricted myelin epitope was hampered by the overexpression of HLA-A*02 molecules, confirming the opposite (and interacting) action of MHC class I-restricted myelin epitopes on EAE onset [124]. Furthermore, it has been hypothesized that the expression of i-proteasome limits the generation of self-epitopes associated with autoimmune responses [38] and we have proposed that a link exists between a genetic protection toward MS and an i-proteasome polymorphism that impairs the generation of a specific MBP epitope [22]. We therefore conclude that i-proteasomes could play a role in the CD8^+^ T cell-mediated immune response in MS, and further studies shall better define the role of CD8^+^ T cells in this pathology and identify which epitopes trigger a deleterious autoimmune CD8^+^ T cell reaction and how they are generated by different proteasome isoforms.

## Figures and Tables

**Table 1:** Proteasome isoforms as potential targets of immunological pathways. The table summarizes the major results that may help future studies to understand how the inhibition of distinct proteasome isoforms may affect the development and progression of MS.

Final targets^a^	Anatomic area	Proteasome subunits inhibited^b^	MS forms	Potential effects^c^	References
CD8^+^ T cells	Thymus, lymph nodes, CNS	*β*1i, *β*2i, *β*5i	NA	+/−	[22, 29, 38, 123, 124]
CD4^+^ Th17 cells	Thymus, lymph nodes, CNS, gut	*β*1i, *β*2i, *β*5i	NA	+	[51, 67, 68, 70, 72, 73]
B cells	Thymus, lymph nodes, CNS	*β*1i	RR, PP, SP	+	[80, 81, 85]
Proteasome Abs	Serum	NA	RR, PP, SP	NA	[90]
Circulating proteasome	Serum	NA	NA	NA	[94, 99]
CNS parenchyma	CNS	*β*5i	NA	−	[31]

^a^Pathways that are directly or indirectly affected by treatment with proteasome inhibitors; ^b^evidence from studies where the inhibition/depletion of specific proteasome subunit provided hints about their potential effect on MS; ^c^these effects also include speculative arguments on how the proteasome subunit inhibition may affect specific pathways. The detrimental or beneficial effects are marked as “−” or “+,” respectively; NA = not available evidence.

## Abbreviations

APC: Antigen presenting cells
AutoAbs: Autoantibodies
BBB: Blood-brain barrier
CNS: Central nervous system
CSF: Cerebrospinal fluid
DCs: Dendritic cells
EAE: Experimental autoimmune encephalitis
FoxP3: Forkhead box P3
IFN: Interferon
Ig: Immunoglobulin
IL: Interleukin
I-proteasome: Immunoproteasome
MHC: Major histocompatibility complex
MBP: Myelin basic protein
MOG: Myelin oligodendrocyte glycoprotein
MS: Multiple sclerosis
PPMS: Primary progressive MS
PCPS: Proteasome-catalyzed peptide splicing
RRMS: Relapsing-remitting MS
SPMS: Secondary progressive phase MS
S-proteasome: Standard proteasome
TcR: T cell antigen receptor
Th: T helper cells
TNF-*α*: Tumor necrosis factor-*α*
Treg: Regulatory T cells
UPS: Ubiquitin-proteasome system.
